# Staging of oral cavity cancer in the 8th edition of the TNM classification: The role of computed tomography in the assessment of depth of invasion and extranodal extension^☆^^☆☆^

**DOI:** 10.1016/j.bjorl.2025.101645

**Published:** 2025-06-04

**Authors:** Leticia de Franceschi, João Manoel Miranda Magalhães Santo, Anniely Mourão de Abreu, Marco Aurélio Vamondes Kulcsar, Claudio Roberto Cernea, Marcio Ricardo Taveira Garcia, Rogerio Aparecido Dedivitis, Leandro Luongo Matos

**Affiliations:** a Universidade de São Paulo (USP), Faculdade de Medicina (FM), Hospital das Clínicas (HC), São Paulo, SP, Brazil; b Universidade de São Paulo (USP), Faculdade de Medicina (FM), Hospital das Clínicas (HC), Instituto de Radiologia (InRad), São Paulo, SP, Brazil; c Instituto do Câncer do Estado de São Paulo (ICESP), São Paulo, SP, Brazil

**Keywords:** Mouth, Mouth neoplasms, Neoplasm staging, Lymphatic metastasis

## Abstract

**Objective:**

Suggest and validate pre-surgical tomographic evaluation criteria for OCC based on the pathological DOI criteria.

**Methods:**

A retrospective cohort study was conducted with 80 patients with tongue and floor of mouth tumors, T1 and T2, followed from 2009 to 2015. Patients were initially classified according to the 7th edition of the TNM (TNM-7) using pathological samples and then reclassified based on the updated 8th edition of the TNM (TNM-8) criteria. The same was done radiologically, using extrapolation and adaptation of the pathological criteria to tomographic evaluation. The results of pathological staging were compared and correlated with the main clinical outcomes. The same was done with the radiological results. To evaluate the pre-surgical accuracy of tomographic staging, radiological and pathological results were compared.

**Results:**

Regardless of the criteria (TNM-7 or TNM-8), it was observed that Computed Tomography (CT) with venous contrast may be used to guide the initial staging. Strong positive correlation was observed between tumor thickness and DOI at both CT and pathology. Considerable upstaging was observed in the sample of assessed patients for both criteria. In the comparison between tomographic and pathological T classifications, moderate correlation was observed in relation to thickness and DOI.

**Conclusion:**

Computed tomography can be used to evaluate the depth of preoperative invasion in oral cavity tumors according to the criteria established in the present study.

**Level of evidence:**

Level III.

## Highlights

• Strong positive correlation between tumor thickness and tomographic depth of invasion.
• Positive correlation between tomographic tumor thickness and pathological tumor thickness.
• Positive correlation between tomographic depth of invasion and pathological depth of invasion.
• Tomographic evaluation of deep of invasion upstaged most of lesions.

## Introduction

In 2016, the American Joint Committee on Cancer (AJCC) published a review of the Tumor, Nodule and Metastasis cancer staging system (TNM-8).^1^, ^2^

Depth of Invasion (DOI) is already assessed in the AJCC Cancer Staging System for other types of cancer, such as melanoma and cutaneous and uterine cervix Squamous Cell Carcinoma (SCC). Recently, this factor was identified as an independent predictor of recurrence and survival in oral SCC,^3^ and was incorporated into this edition.

Oral Cavity Cancer (OCC) staging includes the evaluation of the tumor DOI in both clinical assessment and postoperative pathological outcome, but the clinical evaluation of DOI is not well defined, as well as the determination of this measure by imaging examinations such as Computed Tomography (CT). Presence of Extranodal Extension (ENE) has also been included in this edition of the TNM cancer staging system, and should be assessed clinically, through imaging examinations, and in anatomopathological study. This factor is considered one of the most important prognostic predictors in Head and Neck (HN) cancer.^4^

The objective of the present study was to suggest pre-surgical tomographic evaluation criteria from the new pathological criteria of the TNM-8, based on comparative assessment between the pathological and radiological stages.

## Methods

This is a retrospective, cohort study approved by the Research Ethics Committee (protocol nº 228/14) of the aforementioned Institution conducted with 80 consecutive patients with Squamous Cell Carcinoma (SCC) of the tongue or floor of mouth, T1 or T2, submitted to surgical treatment with curative intent from August 2009 to December 2015. Exclusion criteria comprised patients who had undergone rescue surgeries and who did not have CT after contrast injection.

Clinical and demographic data were obtained by reviewing the patients’ electronic medical records. The following data were collected: gender, age, smoking/alcoholism, subsite and characteristics of the primary lesion, lymph node status, presence of distant metastasis, and pathological data, resection margins, degree of differentiation, presence of perineural or angiolymphatic invasion, tumor dimensions, tumor thickness, depth of invasion, lymph node metastases, and extracapsular spread.

Pathological parameters were analyzed by a single pathologist. Depth of Invasion (DOI) was measured from the epithelial basement membrane to the deepest point of tumor invasion in all cases, and stratified as 5 and 10 mm for analysis, as established in other studies with the same cohort^5^, ^6^ and according to the AJCC cancer staging manual recommendation for Oral Cavity Carcinoma (OCC). Extranodal Extension (ENE) was considered in a metastatic lymph node when extent of the metastatic carcinoma was observed through the capsule into the surrounding connective tissue.^4^

**Fig. 1:**
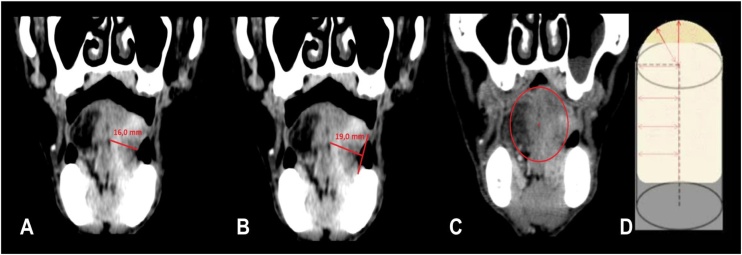
Demonstration of the method used to measure tumor thickness and depth of invasion by computed tomography (coronal images) proposed by the AJCC Cancer Staging System. (A) Demonstration of the method used to measure tumor thickness: measurement used in the old version of TNM classification for oral cavity cancers (7th edition); (B) Demonstration of the method used to measure the depth of invasion (extrapolation of the standardized method in anatomopathological samples by AJCC described at the new TNM ‒ 8th edition). The measurement is based on the conic format of the tongue; (C) An imaginary line is drawn between the two edges of the intact mucosa; (D) Another line is drawn from the point of greatest invasion to the first line, taking as reference the central point of the tongue, the latter, then, will be the measure of depth of invasion).

Tumors were also staged radiologically (T and N radiologic staging) from a pioneering extrapolation of the pathological criteria of ENE and DOI, using a CT study with intravenous contrast (Fig. 1).

For the T-stage, the lesion is initially identified, which usually presents greater enhancement than normal peripheral tissue, and then its depth is dimensioned. Measurement is based on the conic format of the tongue, and depth is measured from an imaginary line drawn at the level of the nearest intact squamous mucosa to the point of greatest invasion, a line guided by the cone radius.

Classification of the T-image was analyzed with extrapolation of the pathological DOI model (measured from the outer-line level of the nearest adjacent normal mucosa), using contrast-enhanced CT.

For the N stage, characteristics of extracapsular spread, such as infiltration of adjacent fatty and muscular planes and irregularity of the capsular contour are considered.

### Statistical analysis

Values obtained through the study of each quantitative variable were organized and described as mean, standard deviation, and minimum and maximum. Absolute and relative frequencies were used for analysis of the qualitative variables. Frequency comparisons were performed using the Chi-Squared or Fisher’s Exact tests. Variables were classified as parametric by the Kolmogorov-Smirnov test and correlations between the independent quantitative variables were determined by the Pearson’s correlation test. The SPSS 17.0 (SPSS Inc; Ilinois, USA) software was used to process the data. A significance level of 5% (*p* ≤ 0.05) was adopted for all statistical analyses.

## Results

The sample was composed of 80 patients with Squamous Cell Carcinoma (SCC) of the tongue and floor of mouth at stages such as T1 or T2 according to the AJCC Cancer Staging System manual ‒ 7^th^ edition.^7^ The sample preferably included male individuals, smokers and alcoholics, aged approximately 60–80 years (Table 1).

**Table 1:** Descriptive data of the study sample.

Variables	Results
Demographic data	
Male gender	62 (77.5%)
Age (in years)	60.0 ± 10.3 (34–87)
Subsite of primary lesion	
Oral tongue	45 (56.2%)
Floor of mouth	35 (43.8%)
Smoking	70 (87.5%)
Alcohol abuse	56 (70.0%)
*Anatomopathological data*	
Free resection margins	78 (97.5%)
Degree of differentiation	
Well differentiated	28 (35.0%)
Moderately differentiated	49 (61.3%)
Poor differentiated	3 (3.8%)
Perineural invasion	31 (38.8%)
Angiolymphatic invasion	13 (16.3%)
Tumor size^a^	23.2 ± 12.3 (1–67) mm
Tumor thickness^a^	11.4 ± 8.5 (1–39) mm
Depth of invasion^a^	11.3 ± 8.4 (1–39) mm
Lymph node metastases	33 (41.3%)
Extracapsular spread (*n* = 25)	12 (48.0%)

a Mean ± Standard deviation (minimum–maximum).

**Fig. 2:**
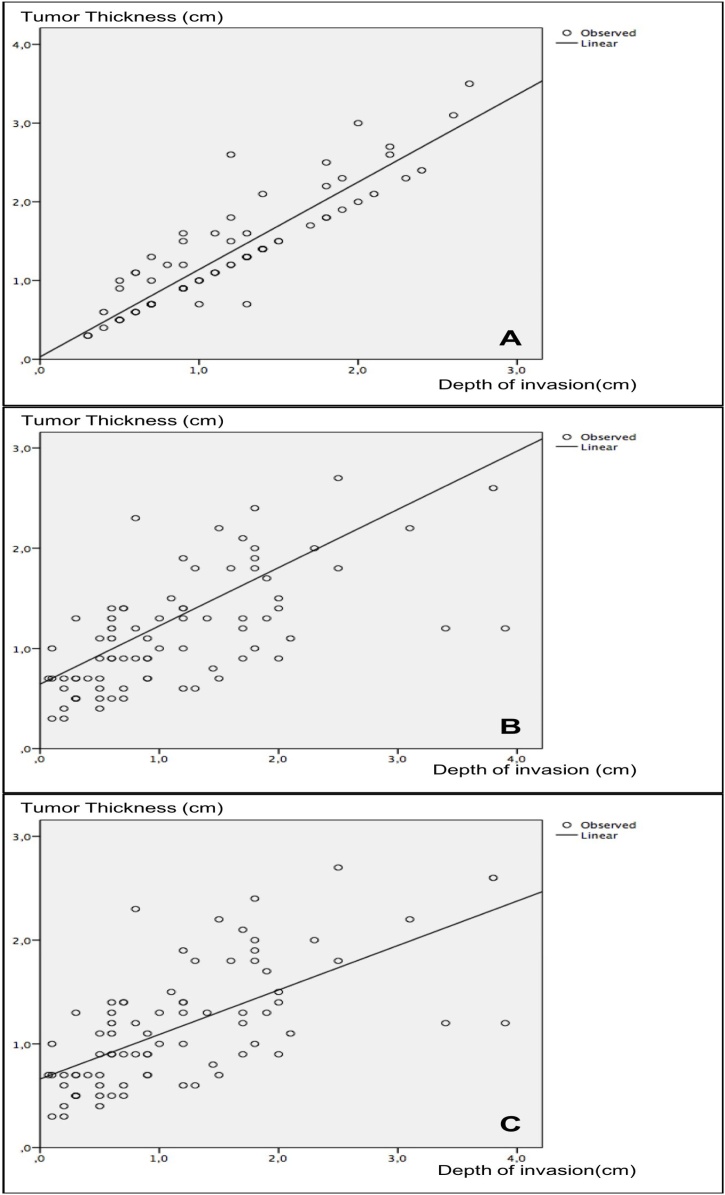
Scatter plots for correlation: (A) Strong positive correlation between tumor thickness and tomographic depth of invasion (*r* = 0.905; *p* < 0.001 ‒ Pearson’s correlation test); (B) Positive correlation between tomographic tumor thickness and pathological tumor thickness (*r* = 0.703; *p* < 0.001 ‒ Pearson’s correlation test); (C) positive correlation between tomographic depth of invasion and pathological depth of invasion (*r* = 0.693; *p* < 0.001 ‒ Pearson’s correlation test).

Staging of all patients was reviewed according to the guidelines of the AJCC Cancer Staging System ‒ 8th edition.^1^ Tumor thickness and Depth of Invasion (DOI) were tomographically measured and histologically revised. Results were as follows: mean tomographic tumor thickness of 13.1 ± 7.0 (minimum of 3 and maximum of 35 mm) and DOI of 11.5 ± 5.7 (minimum of 3 and maximum of 27 mm). Strong positive correlation was found between tumor thickness and tomographic DOI (*r* = 0.905; *p* < 0.001 ‒ Pearson’s correlation test) and between tumor thickness and pathological DOI (*r* = 0.998; *p* < 0.001 ‒ Pearson’s correlation test). Moderate positive correlation was also observed between tomographic tumor thickness and pathological tumor thickness (*r* = 0.703; *p* < 0.001 ‒ Pearson’s correlation test) and between tomographic DOI and pathological DOI (*r* = 0.693; *p* < 0.001 ‒ Pearson’s correlation test). Fig. 2 shows the scatter plots between the referred correlations. It is worth mentioning that, if pathological tumor thickness were used as a parameter for the new staging instead of DOI, as suggested in the new AJCC manual, only four (5.0%) patients would have been incorrectly classified, which is justified by the high correlation between the two measures (Fig. 2B); however, this rate would have been 15% (12 cases) if both measurements were performed through CT.

T-clinical stages were then compared between the two editions of the AJCC Cancer Staging System manual (Table 2). Fifty-two patients (65.0%) received upstaging when tomographic DOI was added to the T-classification.

**Table 2:** Comparison between the T-clinical stages.

	T Classification (AJCC Cancer Staging System ‒ 8th edition)					Total
		T1	T2	T3	T4a	
T classification (AJCC 8th edition)	T1	5 (23.8%)	11 (52.4%)	5 (23.8%)	0 (0.0%)	21 (26.3%)
	T2	1 (1.7%)	23 (39.0%)	35 (59.3%)	0 (0.0%)	59 (73.8%)
	Total	4 (5.0%)	34 (42.5%)	42 (52.5%)	0 (0.0%)	80 (100.0%)

Note: The main groups that received upstaging are shown in bold; p = 0.023 (Chi-Squared test).

Coincidence between the new clinical classification and the pathological stage was 52.5%, or 42 cases (Table 3), especially in T3 cases.

**Table 3:** Comparison between the T-clinical and pathological stages.

	pT Classification (AJCC Cancer Staging System ‒ 8th edition)					Total
		pT1	pT2	pT3	pT4a	
T classification (AJCC 8th edition)	T1	4 (66.7%)	2 (33.3%)	0 (0.0%)	0 (0.0%)	6 (7.5%)
	T2	13 (38.2%)	12 (35.3%)	9 (26.5%)	0 (0.0%)	34 (42.5%)
	T3	1 (2.5%)	11 (27.5%)	**28 (70.0%)**	0 (0.0%)	40 (50.0%)
	Total	18 (22.5%)	25 (31.3%)	37 (46.3%)	0 (0.0%)	80 (100.0%)

Note: The main group of coincidence between the two classifications is shown in **bold**; *p* = 0.001 (Chi-Squared test).

N-clinical stages were also compared between the two editions of the AJCC Cancer Staging System manual (Table 4). Eleven (13.8%) patients received upstaging when signs of extracapsular spread were evidenced by CT. Coincidence between the new clinical classification and the pathological stage was 55.5%, or 44 cases (Table 5), especially in N0 and N3b cases. Nevertheless, no statistically significant difference was found between the tomographic evidence of extracapsular spread to CT and the definitive anatomopathological examination (28.6% of extracapsular spread in patients without this characteristic to CT vs. 58.3% for those with signs of extracapsular spread to image examination; p = 0.142 ‒ Fisher’s exact test).

**Table 4:** Comparison between the N-clinical stages.

		N Classification (AJCC Cancer Staging System ‒ 8th edition)							Total
		N0	N1	N2a	N2b	N2c	N3a	N3b	
N Classification (AJCC 7th edition)	N0	46 (100.0%)	0 (0.0%)	0 (0.0%)	0 (0.0%)	0 (0.0%)	0 (0.0%)	0 (0.0%)	46 (57.5%)
	N1	0 (0.0%)	15 (88.2%)	**2 (11.8%)**	0 (0.0%)	0 (0.0%)	0 (0.0%)	0 (0.0%)	17 (21.3%)
	N2a	0 (0.0%)	0 (0.0%)	1 (100.0%)	0 (0.0%)	0 (0.0%)	0 (0.0%)	0 (0.0%)	1 (1.3%)
	N2b	0 (0.0%)	0 (0.0%)	0 (0.0%)	4 (40.0%)	0 (0.0%)	0 (0.0%)	**6 (60.0%)**	10 (12.5%)
	N2c	0 (0.0%)	0 (0.0%)	0 (0.0%)	0 (0.0%)	3 (50.0%)	0 (0.0%)	**3 (50.0%)**	6 (7.5%)
	Total	46 (57.5%)	15 (18.8%)	3 (3.8%)	4 (5.0%)	3 (3.8%)	0 (0.0%)	9 (11.3%)	80 (100.0%)

Note: The main groups that received upstaging are shown in **bold**; p < 0.001 (Chi-Squared test).

**Table 5:** Comparison between the N-clinical and pathological stages.

		N Classification (AJCC Cancer Staging System ‒ 8^th^ edition)							Total
		N0	N1	N2a	N2b	N2c	N3a	N3b	
N Classification (AJCC 7th edition)	N0	**37 (**80.4%**)**	2 (4.3%)	1 (2.2%)	4 (8.7%)	1 (2.2%)	0 (0.0%)	1 (2.2%)	46 (57.5%)
	N1	8 (53.3%)	2 (13.3%)	2 (13.3%)	1 (6.7%)	1 (6.7%)	0 (0.0%)	1 (6.7%)	15 (18.8%)
	N2a	0 (0.0%)	1 (33.3%)	0 (0.0%)	0 (0.0%)	0 (0.0%)	0 (0.0%)	2 (66.7%)	3 (3.8%)
	N2b	1 (25.0%)	0 (0.0%)	0 (0.0%)	1 (25.0%)	0 (0.0%)	0 (0.0%)	2 (50.0%)	4 (5.0%)
	N2c	1 (33.3%)	0 (0.0%)	0 (0.0%)	1 (33.3%)	1 (33.3%)	0 (0.0%)	0 (0.0%)	3 (3.8%)
	N3a	0 (0.0%)	0 (0.0%)	0 (0.0%)	0 (0.0%)	0 (0.0%)	0 (0.0%)	0 (0.0%)	0 (0.0%)
	N3b	0 (0.0%)	0 (0.0%)	1 (11.1%)	2 (22.2%)	2 (22.2%)	0 (0.0%)	**4 (**44.4%**)**	9 (11.3%)
	Total	47 (58.8%)	5 (6.3%)	4 (5.0%)	9 (11.3%)	5 (6.3%)	0 (0.0%)	10 (12.5%)	80 (100.0%)

Note: The main group of coincidence between the two classifications is shown in **bold**; *p* < 0.001 (Chi-Squared test).

## Discussion

Since its first publication in 1977, the Tumor, Nodule, and Metastasis (TNM) staging of Squamous Cell Carcinoma (SCC) of the oral cavity, through the AJCC Cancer Staging System manuals, has remained essentially the same.^8^

Given the simplicity of the existing AJCC manuals, this system has offered clinical accessibility for estimating patient survival. However, several studies have demonstrated that only TNM staging is insufficient to determine disease prognosis in many cases. Thus, other clinical, anatomopathological and molecular characteristics have shown association with prognosis in several studies. Consequently, new factors have been added to this classification aiming at greater prediction of outcomes.

Depth of invasion is a well-established parameter in the AJCC Cancer Staging System for other types of malignant neoplasms, such as melanoma, cutaneous squamous cell carcinoma, and mucosal malignancies of the cervix, esophagus, stomach and colon. Several research groups have suggested including DOI in oral cavity cancer classification, which was implemented at the end of 2016 with the publication of the 8th edition of the AJCC Cancer Staging System manual. In addition, DOI is a well-established parameter of recurrence and survival for this type of cancer.^6^, ^9^, ^10^

It is also known that Computed Tomography (CT) and Magnetic Resonance Imaging (MRI) are the most significant imaging methods in preoperative staging of head and neck tumors because they provide information on lesion extension, infiltration of large vessels, and lymph node metastasis, thus facilitating the planning and prognosis of treatment.^11^, ^12^, ^13^

The present study evaluated and compared staging of SCC of the oral cavity using the T and N classifications of the 7th and 8th editions of the AJCC Cancer Staging System manual through CT (T and N), and then compared the results with those of the new pathological stages (pT and pN) of these same editions ‒ a pioneering study in this field.

Strong positive correlation was found between tumor thickness and DOI in both tomography and pathology, justified by the overlapping of thickness and depth measurements (identical in most cases).

Considerable upstaging was observed in the sample of patients investigated, which can be justified by the greater sensitivity of the values adopted in the new T-staging, whose cut-off point between a T1 tumor and a T2 tumor was 2.0 cm (regarding thickness) and 0.5 cm (with respect to depth). Of the 21 patients classified as T1 at the TNM-7, 16 (76.2%) received upstaging at the TNM-8, representing the group with the highest divergence between the editions.

In the comparison between tomographic and pathological T-staging, moderate correlation was observed in both tumor thickness and depth.

Upstaging in the study sample was more evident in the clinical assessment compared with that in the pathological analysis, raising a question about the difficulty in measuring the tomographic examination and the reduction of thickness and DOI during histological processing.

The difficulties encountered in the evaluation of tomographic T-stage were associated with very early lesions ‒ often dependent on previous clinical evaluation to guide the identification in the images; with densification of the adipose/locoregional inflammatory reaction, that overestimated the measurements; and with occurrence of beam-hardening artifacts. The latter are generated close to high density bodies (such as dental metal implants, intraradicular nuclei, and crowns), which are quite prevalent in the general population, as well as in the sample investigated.^14^

## Conclusion

Regardless of the new evaluation criteria, Computed Tomography (CT) with venous contrast can be used to guide initial staging and treatment in oral cavity cancers. However, further studies are necessary to determine the values of tomographic depth of invasion equivalent to the pathological sections already studied and now incorporated into the AJCC Cancer Staging System ‒ 8th edition.

## Financial support

None.

## Declaration of competing interest

The authors declare no conflicts of interest.
